# Validity and Reliability of a Commercially Available Indoor Tracking System to Assess Distance and Time in Court-Based Sports

**DOI:** 10.3389/fpsyg.2019.02076

**Published:** 2019-09-10

**Authors:** Enrique Colino, Jorge Garcia-Unanue, Javier Sanchez-Sanchez, Javier Calvo-Monera, Manuel Leon, María Jose Carvalho, Leonor Gallardo, Jose Luis Felipe, Archit Navandar

**Affiliations:** ^1^IGOID Research Group, University of Castilla–La Mancha, Toledo, Spain; ^2^Faculty of Sport Sciences, Universidad Europea de Madrid, Madrid, Spain; ^3^Nothingbutnet S.L., Valencia, Spain; ^4^Faculdade de Desporto da Universidade do Porto, Porto, Portugal

**Keywords:** team sports, player tracking, indoor tracking, training load, match analysis

## Abstract

The aim of this study was to evaluate the validity and reliability of a commercially available local position measurement (LPM) tracking system when assessing distance and running time at different speeds. Fifteen male healthy athletes performed 15 m displacements at walking, running and sprinting speed. Data recorded by the LPM system were compared to those from the reference equipment, consisting of measuring tape and electronic timing gates placed at 0, 5, 10, and 15 m. Mean error, mean absolute error (MAE), standard deviation (SD) of the measurement error, maximum measurement error and root mean square error (RMSE) were calculated to determine the validity for distance and the running time variables. Product-moment correlation and intraclass correlation coefficient (ICC) were also used for the running time. Finally, the reliability of the distance was carried out comparing data from the three repetitions with the standard tape measure using a linear mixed model and the typical error as mean coefficient of variation (CV) (%). MAE shows errors under 0.18 m for the distance variable at all speeds and under 0.08 s for the running time variable at all speeds, except from 15 m at walking. Product-moment correlations were high to nearly perfect for running time (range: 0.60–0.99), ICC varied between high (0.75–0.90) and extremely high (>0.99) for most measures, and coefficients of variation remained almost invariable as speed increased (walking: 2.16; running: 2.52; sprinting: 2.20). The tested LPM system represents a valid and reliable method for monitoring distance during different constant speeds over a straight line, as long as there is no signal loss. However, the running time errors could be too large for performance tests that require acute precision.

## Introduction

Accurate assessment of athletes’ movement profile during training and match play in team sports presents useful information that could assist coaches and trainers in the development of specific conditioning activities and recovery strategies (Duffield et al., 2010). Initially, computer-based tracking systems were used for assessing players at constant running speeds such as distance covered and time spent during sports-specific tasks (Edgecomb and Norton, 2006). However, this analysis technique required large amounts of time and was overall difficult to implement when simultaneously assessing large groups of athletes or entire teams. In the last few years, advancements in technology have led to the introduction and rapid proliferation of sophisticated systems such as multiple camera semi-automatic systems, global positioning systems (GPS) and local position measurement (LPM) systems. These technologies are capable of quickly recording and processing the players’ physical exertions throughout an entire match or training session (Carling et al., 2008).

The development of these technologies has mostly been linked with outdoor sports such as football which has seen the rapid evolution of multiple camera and GPS systems, bringing forth a significant improvement in sport performance and player management, thereby contributing to the emergence of a whole new industry. The main contribution of these technologies is that they allow the concurrent analysis of running speeds of numerous players to be completed on a routine basis with affordable expenses (Buchheit et al., 2014).

Computerized analysis using multiple camera, semi-automatic, video match-analysis systems has proved to be a valid and reliable tool to measure player movements and actions, and to classify the game events in professional competitions (Di Salvo et al., 2007, 2008; Carling et al., 2008). However, installing and using this technology generally requires a large investment in economic terms that represents a limitation in most sports.

Global positioning systems devices have also been widely assessed for their validity and reliability in measuring distance, time, speed and acceleration during different sporting actions over the last decade (Edgecomb and Norton, 2006; Barbero-Alvarez et al., 2010; Coutts and Duffield, 2010; Coutts and Duffield, 2010; Gray et al., 2010; Jennings et al., 2010; Waldron et al., 2011; Varley et al., 2012; Vickery et al., 2014), and it has led to GPS being the preferred technology used in player tracking. However, the main limitation of GPS technology is that it requires an “open” sky without obstructions above athletes that ensures clear space for positional data acquisition from satellites. This constraint prevents GPS devices from being used in indoor sports such as basketball or handball. Thus, other technologies such as LPM have been used for the assessment of athletes’ movement profile indoors.

The LPM technology has seen moderate improvements over the last few years, resulting in better stability and precision. The declared relative position measurement accuracy of some of these systems is around 3 cm which can be measured up to 1 kHz (Buchheit et al., 2014). This technology consists of radio systems based on a Bluetooth Low Energy (BLE) radio signals which provide location information through a network of antennas (base stations) and transponders. These base stations are positioned around the playing area to be measured while the transponders are worn by the athletes.

There is limited information regarding the validity and reliability of commercially available indoor tracking systems using LPM technology for assessing constant running speeds during indoor team sports (Frencken et al., 2010; Siegle et al., 2013; Link et al., 2018; Linke et al., 2018). Although LPM systems have been reported to offer higher validity for measuring athletes’ position compared to video and GPS technology (Linke et al., 2018), they still have the highest deviations when measuring distance, which might also cause relevant limitations for the assessment of sprint performance especially over short distances (Link et al., 2018). In the past few years, this type of positioning system has experienced an increasing commercialization, and some of them are currently being used by different basketball teams of the highest category in Spain. The present work aims to assess the validity and reliability of distance and running time measured by means of an LPM system at different speeds, providing an initial contribution for the assessment of this technology.

## Materials and Methods

### Experimental Approach to the Problem

Participants performed repeated straight-line displacements at different speeds while distance and time were assessed using the LPM system and compared to the reference values. Actual distance was measured using a calibrated tape measure and time was measured using four pairs of dual-beam photocells (Witty, Microgate, Bolzano, Italy) placed at the starting line and every 5 m. This technology has been previously considered as a reference tool in the measurement of distances and time (Buchheit et al., 2014) and is the recommended technology to register accurate and reliable short sprint results in scientific research (Haugen and Buchheit, 2016). The tests were conducted in an indoor sport facility with parquet surface, normally used by a basketball team of the ACB (first division of Spanish basketball).

### Subjects

Fifteen trained male athletes (age: 26 ± 3 years; height: 1.85 ± 0.06 m; weight: 79 ± 3 kg) were randomly chosen from a population of basketball players that volunteered to participate in the study. Participants were informed about the experimental procedures and the potential risks and benefits resulting from participating in the study, and they all signed an institutionally approved informed consent document, as outlined in the Declaration of Helsinki. The study protocol was approved by the Local Ethics Committee.

### Procedures

Each participant completed nine 15-m displacements on a straight line separated by 1 min of passive recovery, following procedures mentioned previously (Jennings et al., 2010). The first three displacements were made at a comfortable walking speed; displacements 4 to 6 were performed running at gentle pace; and the last three displacements were performed sprinting at maximum speed. To expand the scope of the study at different speeds and distances, timing gates were placed at 0, 5, 10, and 15 m. Total time (0–15 m) and times of each of the three 5-m splits (0–5, 5–10, and 10–15 m) were recorded.

For the calculation of the distances traveled in a specific time, the LPM system considered the start time of each displacement as that when the first movement occurred (i.e., the first increase on the velocity curve above zero (Petersen et al., 2009; Jennings et al., 2010). This starting time was associated with the moment that the athlete went through the timing gate placed at 0 m mark. Then, the time from the timing gates was used to indicate the end of the movement for each known distance. Thus, for a time interval determined by two successive cuts of timing gates, distance measured with the LPM system was compared to the current distance between both timing gates.

In addition, the time taken to complete the distance of 15 m (according to the LPM system) was recorded. This data was compared to the time recorded by the timing gates for the same distance. Therefore, distances and times recorded by the LPM system in each test were compared to the taped distances and the times obtained from the timing gates, respectively. Only trials where an accurate measurement was made by both the timing gates (trials discarded for timing gate measurement error: walking = 2, jogging = 4, sprinting = 19) and the LPM system (trials discarded due to momentary drop in signal: walking = 5, jogging = 3, and sprinting = 12) were considered for analysis.

#### LPM System

**FIGURE 1:**
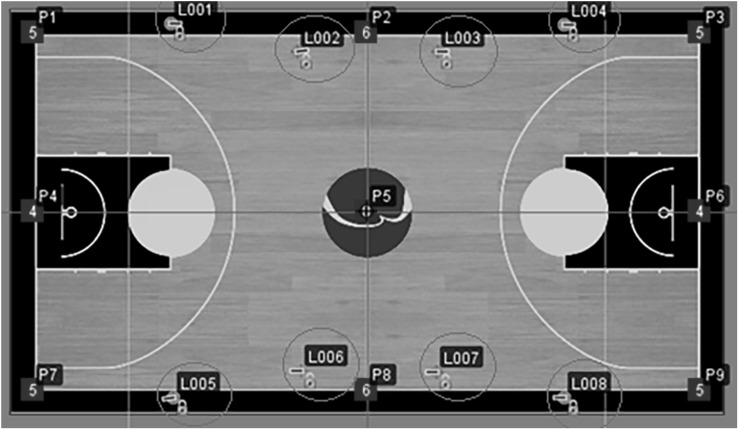
The positioning of the locaters (L) and reference points (P) of the NBN system.

The system used in this study is commercially known as NBN23 (Nothing But Net, Valencia, Spain) and is composed of two different elements. First, the system hardware was created by the Finnish company Quuppa (Nokia Corporation, 2014). This technology consists of locators and the devices that the players carry (tags) which were placed on the waist, at the back of the trousers at the height of the sacrum. This position represents the approximate position of the center of mass of the subject and avoids a possible shielding of the signal with the participant’s own body. Tags are emission devices, and the system works by triangulating the signal they emit. The frequency at which they send information can be varied between 9, 17, 33, and 50 Hz. The frequency band is 2.4 GHz, the system delay is 100 ms with a capacity of 400 information packets per second. In this study, eight locators (firmware v9.047) placed in two groups of four at a height of 13.51 m (Figure 1) were used along with eight different tags at a frequency of 17 Hz (Nokia Technologies Oy, 2014).

Secondly, the specific software for the LPM system was used (Nothingbutnet v1.1.3, Spain, 2016). This software adds a Kalman filter to the position coordinates (given in terms of *X* and *Y*) provided by Quuppa to reduce noise and measurement error with a cut-off frequency of 3 Hz. The distance traveled was calculated through the position changes on the *X* and *Y* axes. To carry out the tests, the participant started from a static position on the free throw line.

### Statistical Analyses

Mean error, mean absolute error (MAE), standard deviation (SD) of the measurement error, maximum measurement error and root mean square error (RMSE) were determined for assessing validity of the distance variable, considering the tape measurement as the standard. A plot with the difference between criterion and LPM distance in each speed and 5-m split (0–5, 5–10, and 10–15 m) was also added.

Validity of the running time variable was performed between the times recorded using the LPM system and the interval times recorded by the timing gates for equal distances. Product-moment correlation (r), intraclass correlation coefficient (ICC), absolute agreement with 95% confidence intervals (Field, 2013) and Bland–Altman plots were used. Moreover, similar to the distance measures, MAE, SD of the measurement error, maximum measurement error and RMSE were determined taking the timing gates data as the standard. Product-moment correlation results were evaluated as trivial (<0.10), small (0.10–0.30), moderate (0.30–0.50), large (0.50–0.70), very large (0.70–0.90), nearly perfect (0.90–1.00) scores (Johnston et al., 2014). ICC were evaluated as very low (<0.20), low (0.20–0.50), moderate (0.50–0.75), high (0.75–0.90), very high (0.90–0.99), and extremely high (>0.99) scores (Buchheit et al., 2014).

The reliability of the distance was carried out comparing data from the three repetitions with the standard tape measure using a linear mixed model. For this model, the repetition was considered as the fixed factor, and the participant was the random factor. Furthermore, typical error as mean coefficient of variation (CV) (%) was calculated by the Hopkins reliability spreadsheet (Hopkins, 2015), and used among the three trials for each velocity (walking, running, and sprinting). Once CV was established, it was divided into criterion categories, being rated as good (CV < 5%), moderate (CV 5 to 10%) or poor (CV > 10%) (Atkinson and Nevill, 1998; Duthie et al., 2003; Johnston et al., 2014).

Normality and homogeneity of the variance were previously tested and assumed by the Kolmogorov Smirnov test and the Levene’s statistical analysis. The level of significance was established at *p* < 0.05. Data were analyzed using SPSS version 21.0 (SPSS Inc., Chicago, IL, United States) and SAS University Edition (SAS Institute Inc., 2015, Cary, NC, United States).

## Results

**FIGURE 2:**
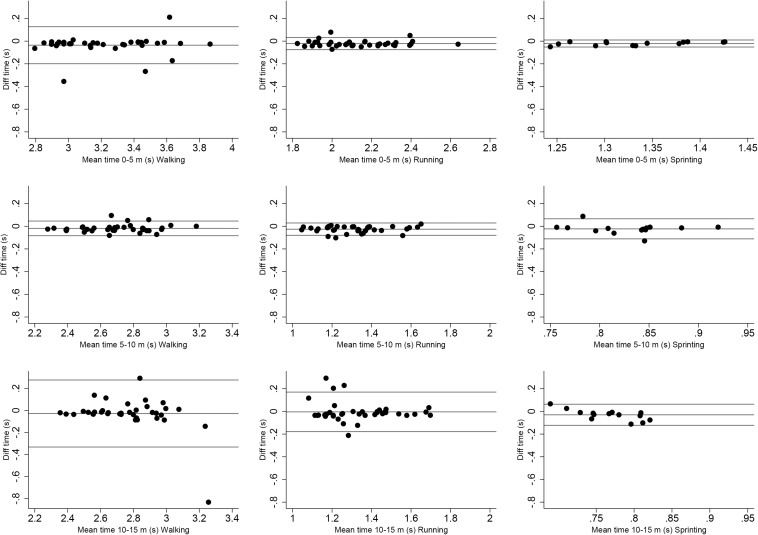
Bland-Altman plots representing the differences between the time measured by the NBN system and the standard timing gates.

**FIGURE 3:**
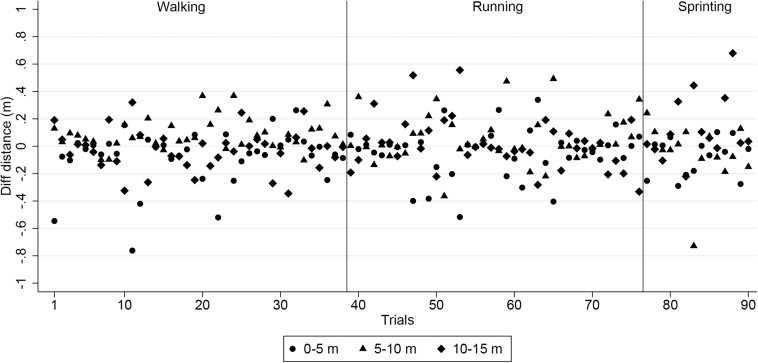
Bland-Altman plots representing the differences between the distance measured by the NBN system and the taped distances.

Tables 1, 2 show the errors measured between the LPS system and the gold standard measurement: the fixed taped distance and the timing gates. The mean error, and the MAE show values under 0.18 m for the distance variable at all speeds, which is lower than 5% of the actual measured value (Figures 2, 3). In case of the time variable a slightly higher value of MAE was recorded at the longer distances. In this case, the ICC went from high to extremely high values at walking and running speeds, while at sprinting speed it presents moderate to very high correlation values. In spite of the lower coefficients at sprinting speed, correlations values are still above the threshold of moderate magnitude.

**TABLE 1:** Validity and agreement of the distance variable.

	***N***	**Mean error (m)**	**MAE (m)**	**SD of measurement error (m)**	**Max error (m)**	**RMSE (m)**
**Walking**						
0–5 m	38	0.08	0.13	0.20	0.76	0.22
5–10 m	38	–0.08	0.10	0.11	0.37	0.14
10–15 m	38	0.03	0.11	0.15	0.34	0.15
0–10 m	38	0.01	0.13	0.19	0.70	0.19
0–15 m	38	0.03	0.12	0.16	0.45	0.16
**Jogging**						
0–5 m	38	0.05	0.13	0.18	0.52	0.19
5–10 m	38	–0.06	0.12	0.18	0.49	0.19
10–15 m	38	–0.02	0.13	0.19	0.56	0.19
0–10 m	38	0.01	0.16	0.21	0.54	0.19
0–15 m	38	–0.01	0.12	0.15	0.34	0.15
**Running**						
0–5 m	14	0.08	0.12	0.14	0.29	0.15
5–10 m	14	0.05	0.15	0.23	0.73	0.23
10–15 m	14	–0.13	0.18	0.24	0.68	0.26
0–10 m	14	0.13	0.15	0.25	0.91	0.27
0–15 m	14	0.00	0.17	0.26	0.70	0.26

**N*, number of trials; MAE, mean absolute error; SD, standard deviation; RMSE, root mean square error; Max error, maximum error; MAE and RMSE were determined with respect to the measured distance.*

**TABLE 2:** Validity and agreement of the time variable.

**Variables**	***N***	**Mean error (s)**	**MAE (s)**	**SD of measured error (s)**	**Max error (s)**	**RMSE (s)**	***r***	**ICC**
**Walking**								
0–5 m	38	−0.04	0.05	0.08	0.36	0.09	0.96	0.97
5–10 m	38	−0.02	0.03	0.02	0.10	0.04	0.99	0.99
10–15 m	38	−0.03	0.07	0.14	0.83	0.16	0.80	0.87
0–10 m	38	−0.06	0.07	0.07	0.38	0.10	0.99	0.99
0–15 m	38	−0.08	0.12	0.14	0.85	0.18	0.97	0.98
**Running**								
0–5 m	38	−0.02	0.03	0.02	0.08	0.03	0.99	0.99
5–10 m	38	−0.03	0.03	0.03	0.10	0.04	0.99	0.99
10–15 m	38	−0.00	0.05	0.07	0.29	0.09	0.88	0.94
0–10 m	38	−0.05	0.05	0.04	0.14	0.06	0.99	0.99
0–15 m	38	−0.05	0.08	0.06	0.29	0.10	0.98	0.99
**Sprinting**								
0–5 m	14	−0.02	0.02	0.02	0.05	0.03	0.97	0.96
5–10 m	14	−0.02	0.04	0.04	0.13	0.50	0.60	0.71
10–15 m	14	−0.03	0.04	0.03	0.11	0.06	0.64	0.59
0–10 m	14	−0.04	0.05	0.04	0.18	0.07	0.77	0.79
0–15 m	14	−0.07	0.08	0.04	0.15	0.09	0.89	0.83

*MAE, mean absolute error; SD, standard deviation; Max error, maximum error; RMSE, root mean square error; *r*, product-moment correlation; ICC, intraclass correlation coefficient.*

Table 3 shows the reliability results of distance data. In this case, all the values fit within the best criterion category (good). Unlike the previous cases, results show a great consistency in all speeds and distances, with mean CVs remaining below 3% for all speeds (walking: 2.16; running: 2.52; sprinting: 2.20).

**TABLE 3:** Reliability of the distance variable (typical error presented as the mean CV).

**Distance interval**	**CV walking (%)**	**CV running (%)**	**CV sprinting (%)**
0–5 m	3.0	2.4	2.3
5–10 m	2.6	4.1	2.3
10–15 m	2.8	3.2	4.3
0–10 m	1.3	2.0	0.6
0–15 m	1.1	0.9	1.5

*CV, coefficient of variation.*

Finally, Table 4 shows the reliability of the differences in distances between the LPM system measurement and the taped measurement. The results show that the differences between the trials only varied for the 0–15 m distance at a walking speed, and inter-participant differences were observed for the 0–10 m walking distance.

**TABLE 4:** Reliability of the differences in distances between the taped distance and the distance measured by the local position measurement (LPM) system.

	**Fixed effects**				**Random effects**	
	***F***	**df1**	**df2**	***p***	**Wald *Z***	***p***
**Walking**						
0–5 m	3.15	2	22.92	0.062	1.90	0.06
5–10 m	1.04	2	35.00	0.366	–1.46	0.14
10–15 m	0.05	2	23.53	0.952	0.57	0.57
0–10 m	2.61	2	22.65	0.096	2.03	0.04^†^
0–15 m	3.51	2	35.00	0.041^∗^	–0.42	0.67
**Jogging**						
0–5 m	0.22	2	21.65	0.803	1.83	0.07
5–10 m	0.12	2	35.00	0.889	–1.88	0.06
10–15 m	0.78	2	35.00	0.468	–0.33	0.74
0–10 m	0.27	2	35.00	0.767	0.17	0.87
0–15 m	1.12	2	22.71	0.344	1.25	0.21
**Running**						
0–5 m	1.13	2	6.67	0.378	1.04	0.30
5–10 m	0.50	2	4.18	0.639	1.26	0.21
10–15 m	0.49	2	11.00	0.625	–0.18	0.86
0–10 m	0.38	2	4.89	0.701	1.65	0.10
0–15 m	0.98	2	6.98	0.422	0.44	0.66

*A linear mixed model was used with the repetition being the fixed effect and the athlete being the random effect. ^∗^Indicates a significant difference between the difference of the measures in the different repetitions. ^†^Indicates a significant difference between participants in different repetitions. All tests were carried out with α = 0.05.*

## Discussion

This paper presents the validity and reliability of an LPM measuring system for distance and running time during different constant speeds over a straight line. The validity of the system, which consisted of a set of tags placed at the sacrum of the player, was measured against taped distances and timing gates.

When assessing distance validity, results show that the LPM system either over or underestimates the actual distance, but the MAE are well under 0.18 m (percentage of differences is less than 2%). The magnitude of this difference were over those declared by Frencken et al. (2010) when assessing accuracy and validity of an equivalent LPM device in smaller, straight line distances (0.2%), but under that declared for GPS devices when assessing distance at different speeds over a 200 m on a straight line (0.8–2.8%) (Gray et al., 2010). It is important to consider that some noise-inducing variables such as a slightly forward position of the device at the beginning or end of the trial, can cause these differences. Furthermore, the SD of measurement error varied from 0.11 to 0.26 m, but this issue was not discussed in these previous studies.

On the other hand, the results of the present study show that the LPM system offers a higher accuracy than that shown by GPS in other studies such as Coutts and Duffield (2010) (3.6–7.1%), Gray et al. (2010) (0.5–9.8%), Duffield et al. (2010) (4.0–30.0%) or Jennings et al. (2010) (9.0–32.4%), although some of those studies consisted of more complex validation protocols including turns and curved movement paths.

Possibly, one of the most frequent uses in these monitoring systems is the quantification of the load as accumulated distance at different speeds, as previous studies refer to these individual movements in team sports (Gray et al., 2010). Therefore, the LPM system analyzed can be used to quantify distances in simple movements with the same or greater precision than those obtained through a system like the GPS. However, more research is needed involving complex circuits and routes that mimic the movement patterns of sports such as basketball, handball or futsal.

When assessing running time spent to cover a certain distance, validity correlation ranged from very large to nearly perfect in walking and running speeds (0.80–0.99), in agreement to those described by Frencken et al. (2010). When assessing running time at sprinting speed the correlation values down to large (0.60–0.97). In relative terms, the percentage of difference varied between 0 and 2.48% at walking and running speeds and between 4.35 and 6.68% at sprinting speed. In addition, the ICC varied between a high (0.75–0.90) and extremely high (>0.99) except in two time-variables at sprinting speed, where it is still moderate (0.50–0.75). However, MAE remain similar in all speeds, which varied between 0.02 and 0.12. In agreement with previous research validating GPS devices, the accuracy of the LPM system decreased slightly as speed increased (Edgecomb and Norton, 2006; Coutts and Duffield, 2010; Duffield et al., 2010; Jennings et al., 2010; Vickery et al., 2014). These differences are once more in line with those described by Frencken et al. (2010) (1.3–3.9%) and are lower than those generally described for speed in GPS devices as in Duffield et al. (2010) (10–30%). Furthermore, the LPM system provides better level of accuracy for measures of running time compared with other tracking devices such as GPS where ICC has been reported to vary from 0.10 to 0.70 (Duffield et al., 2010) or from 0.50 to 0.86 (Vickery et al., 2014).

The reliability of the LPM system when measuring distance traveled during each bout was good (CV < 5%), being this consistent as speed increases from walking to maximal sprint, and this was confirmed by the linear mixed model. The overall reliability of the LPM system data across all trials remains within the ranges that have been described for other LPM systems (0.4–2.0%) (Frencken et al., 2010) or other tracking devices. For example, regarding GPS systems, Barbero-Alvarez et al. (2010) reported CVs of between 1.2 and 1.7% for summated maximal speed and peak speed expressed during 15 and 30-m repeated sprint tests over seven trials. Others such as Coutts and Duffield (2010) reported good-to-moderate CVs for total distance (3.6–7.1%) and peak speed (2.3–5.8%) when assessing validity and reliability of different GPS devices. However, studies assessing reliability of other tracking devices generally report that the higher the speed, the greater the measurement error (Duffield et al., 2010; Gray et al., 2010; Vickery et al., 2014). Contrarily, in the present study better reliability was observed as intensity increases, with the lowest CVs corresponding to the highest speed.

The measurement can be considered as useful for precise measurements when the noise is at least equal to or lower than the smallest worthwhile change (SWC) (Haugen and Buchheit, 2016). The SWC could be defined as 0.2 multiplied by the between-subject SD (Hopkins et al., 2009; Ferioli et al., 2018). In this study the SWC is between 0.01 and 0.06 s in 5 m splits for the time measurement (between 0.8 and 2.75%). The SD of measurement errors were between 0.02 and 0.14 s in the 5 m splits, which could make the random error higher than the differences in performance between players, presenting a limitation of its use in performance tests carried out over short distances. Nevertheless, it can be a useful device in determining accumulated loads in training and games.

Another limitation of this study was that time and distance were only assessed over standard straight-line 15-m displacements and that true peak speed was not measured directly. Quite often, short sprints, changes of direction and brief maximal accelerations of >1 s are completed in team sports. Although previous methodologies were followed to match the start of timing gates and LPM measurement, there are still possible errors because the LPM system may start recording a few ms before the first timing light cut. There is also a spatial limitation since it was possible that in some specific points of the field (bands or corners) the LPM system could measure differently depending on the distance to the system receivers. Finally, this study also has its own measurement error associated with timing gates. Among them, the possible swinging of arms and legs, an error that can reach 0.02 s (Haugen et al., 2014) and errors associated with the height or distance of the photocells (Cronin and Templeton, 2008). Therefore, the scope of this work is limited, and it is possible that important data may be missed due to the protocol design. Future research should examine the efficacy of this system when assessing more complex protocols.

## Conclusion

The tested LPM system represents a valid and reliable method for monitoring distance and running time during constant speeds over a straight line as long as there is no signal loss. Intensity appears to affect the LPM system running time accuracy, being this lower as speed increases. The typical error measured with CV (%) shows a great consistency regardless of speed and distance, with good reliability results. This implies that the LPM system provides similar consistency than other tracking devices such as GPS system and could be useful for training purposes and determinate external workload. However, the random part of measurement error is higher than the SWC, so the LPM could not be a good system to evaluate performance tests carried out at high speeds in short distances.

## Data Availability

All datasets generated for this study are included in the manuscript and/or the supplementary files.

## Ethics Statement

This study was carried out in accordance with the recommendations of the Institutional Research Board of the Universidad de Castilla–La Mancha, Toledo, Spain with written informed consent from all subjects. All subjects gave written informed consent in accordance with the Declaration of Helsinki. The protocol was approved by the Ethics Committee of the Universidad de Castilla–La Mancha, Toledo, Spain.

## Author Contributions

EC coordinated the research, drafted the initial manuscript, and carried out the statistical analyses. JG-U, JF, and AN substantially contributed to the analysis and interpretation of the data. JS-S helped on the conceptualization and design of the study. JC-M, MC, and ML coordinated the data collection process. All authors contributed to the manuscript revision, read, and approved the submitted version.

## Conflict of Interest Statement

JC-M was employed by company Nothingbutnet S.L. The remaining authors declare that the research was conducted in the absence of any commercial or financial relationships that could be construed as a potential conflict of interest.
